# Evidence of Selection in the Ectodysplasin Pathway among Endangered Aquatic Mammals

**DOI:** 10.1093/iob/obac018

**Published:** 2022-07-19

**Authors:** Neus Font-Porterias, Madeline G McNelis, David Comas, Leslea J Hlusko

**Affiliations:** Departament de Medicina i Ciències de la Vida, Universitat Pompeu Fabra, Institut de Biologia Evolutiva (UPF-CSIC), 08003 Barcelona, Spain; Department of Integrative Biology, University of California, Berkeley, 94720 CA, USA; Departament de Medicina i Ciències de la Vida, Universitat Pompeu Fabra, Institut de Biologia Evolutiva (UPF-CSIC), 08003 Barcelona, Spain; Department of Integrative Biology, University of California, Berkeley, 94720 CA, USA; National Research Center on Human Evolution (CENIEH), 09002 Burgos, Spain

**Keywords:** Positive selection, ectodysplasin pathway, aquatic adaptation, endangered mammals

## Abstract

**Synopsis:**

The ectodysplasin pathway has been a target of evolution repeatedly. Genetic variation in the key genes of this pathway (*EDA*, *EDAR*, and *EDARADD*) results in a rich source of pleiotropic effects across ectodermally-derived structures, including teeth, hair, sweat glands, and mammary glands. In addition, a non-canonical Wnt pathway has a very similar functional role, making variation in the *WNT10A* gene also of evolutionary significance. The adaptation of mammals to aquatic environments has occurred independently in at least 4 orders, whose species occupy a wide geographic range (from equatorial to polar regions) and exhibit great phenotypic variation in ectodermally-derived structures, including the presence or absence of fur and extreme lactational strategies. The role of the ectodysplasin pathway in the adaptation to aquatic environments has been never explored in mammalian species. In the present study, we analyze the genetic variation in orthologous coding sequences from *EDA*, *EDAR*, *EDARADD,* and *WNT10A* genes together with ectodermally-derived phenotypic variation from 34 aquatic and non-aquatic mammalian species to assess signals of positive selection, gene-trait coevolution, and genetic convergence. Our study reveals strong evidence of positive selection in a proportion of coding sites in *EDA* and *EDAR* genes in 3 endangered aquatic mammals (the Hawaiian monk seal, the Yangtze finless porpoise, and the sea otter). We hypothesize functional implications potentially related to the adaptation to the low-latitude aquatic environment in the Hawaiian monk seal and the freshwater in the Yangtze finless porpoise. The signal in the sea otter is likely the result of an increased genetic drift after an intense bottleneck and reduction of genetic diversity. Besides positive selection, we have not detected robust signals of gene-trait coevolution or convergent amino acid shifts in the ectodysplasin pathway associated with shared phenotypic traits among aquatic mammals. This study provides new evidence of the evolutionary role of the ectodysplasin pathway and encourages further investigation, including functional studies, to fully resolve its relationship with mammalian aquatic adaptation.

**Spanish:**

La vía de la ectodisplasina ha sido objeto de la evolución repetidamente. La variación genética en los principales genes de esta vía (*EDA*, *EDAR* y *EDARADD*) da como resultado una gran diversidad de efectos pleiotrópicos en las estructuras derivadas del ectodermo, incluidos los dientes, el cabello, las glándulas sudoríparas y las glándulas mamarias. Además, una vía wnt no canónica tiene un papel funcional muy similar, por lo que la variación en el gen *WNT10A* también tiene importancia evolutiva. La adaptación de los mamíferos a los entornes acuáticos se ha producido de forma independiente en al menos cuatro órdenes, cuyas especies ocupan un amplio rango geográfico (desde regiones ecuatoriales a polares) y presentan una gran variación fenotípica en las estructuras derivadas del ectodermo, incluyendo la presencia o ausencia de pelaje y estrategias de lactancia muy diferentes. El papel de la vía de la ectodisplasina en la adaptación a entornos acuáticos no se ha explorado nunca en especies de mamíferos. En este estudio, analizamos la variación genética en las secuencias codificantes ortólogas de los genes *EDA*, *EDAR*, *EDARADD* y *WNT10A* junto con la variación fenotípica derivada del ectodermo de 34 especies de mamíferos acuáticos y no acuáticos para evaluar señales de selección positiva, coevolución gen-rasgo y convergencia genética. Nuestro estudio revela señales de selección positiva en regiones de las secuencias codificantes de los genes *EDA* y *EDAR* en tres mamíferos acuáticos en peligro de extinción (la foca monje de Hawái, la marsopa lisa y la nutria marina). Estas señales podrían tener implicaciones funcionales potencialmente relacionadas con la adaptación al entorno acuático de baja latitud en la foca monje de Hawái y el agua dulce en la marsopa lisa. La señal en la nutria marina es probablemente el resultado de una mayor deriva genética tras un intenso un cuello de botella y una reducción de la diversidad genética. A parte de selección positiva, no hemos detectado señales sólidas de coevolución gen-rasgo o cambios convergentes de aminoácidos en la vía de la ectodisplasina asociados a rasgos fenotípicos compartidos entre mamíferos acuáticos. Este estudio proporciona nuevas evidencias del papel evolutivo de la vía de la ectodisplasina y quiere promover futuras investigaciones con estudios funcionales para acabar de resolver la relación de esta vía con la adaptación acuática de los mamíferos.

## Introduction

The genetic mechanisms that structure phenotypic variation significantly influence the ability of an evolutionary lineage to move into and exploit a novel environment. Ultimately, the genetic variation within that mechanism determines whether or not that lineage will thrive and diversify in the new niche. Almost 30 years of research reveals the important role in evolution played by the ectodysplasin pathway, a member of the Tumor necrosis factor (TNF) family of ligands and receptors. There are three major genes in the ectodysplasin pathway, *Ectodysplasin A* (*EDA*), the *Ectodysplasin A Receptor* (*EDAR*), and the *Ectodysplasin A Receptor Death Domain* (*EDARADD*). Whereas many signaling pathways (such as Notch, Wnt, and Hedgehog) have strong pleiotropic effects across numerous tissue types at numerous stages of embryonic development, the ectodysplasin pathway's effects are specific to ectodermal appendages (reviewed by (Sadier et al. 2014)). A decrease in ectodysplasin activity generally leads to a reduction in dental formula, tooth shape, scales, feathers, hair, mammary glands, salivary glands, and sweat glands whereas an increase in ectodysplasin activity leads to an increase in these same anatomical structures (Sadier et al. 2014).

Evolution has occurred along the variation associated with the ectodysplasin pathway repeatedly over short evolutionary time frames. For example, variation in bird feathers is patterned by *EDA* expression's influence on *the Fibroblast Growth Factor 20* (*FGF20*; (Ho et al. 2019)). Stickleback fish have repeatedly gained bony armor as they moved from marine into freshwater lake environments at the end of the last ice age, with a variant of *EDA* being the causal mutation (Des Roches et al. 2020: 202; Schluter et al. 2021). Other fish have also exploited the ectodysplasin pathway, such as Tibetan snow trout (Zhang et al. 2018) and African cichlids (Fraser et al. 2009). Mice have long been a model for *EDA* deficiencies (Srivastava et al. 1997), and the evolution of murine dentitions can be recapitulated by gradually increasing the ectodysplasin a protein added to tooth explants (Harjunmaa et al. 2014). Even in recent human evolution we see that a variant of the *EDAR* gene experienced a bout of intense selection during the last ice age (Sabeti et al. 2007; Kamberov et al. 2013), possibly related to adaptation to the extremely low ultraviolet radiation environment of the Arctic (Hlusko et al. 2018: 201).

Genome wide association studies in humans perhaps reveal the pleiotropic effects of the ectodysplasin pathway most clearly. Genetic variation in *EDA*, *EDAR*, and *EDARADD* is associated with normal and pathological variation across epithelial structures, including hair, sweat and sebaceous glands, mammary glands, ears, and teeth (Fujimoto et al. 2008; Chang et al. 2009; Kimura et al. 2009; Park et al. 2012; Kamberov et al. 2013; Lindfors et al. 2013; Tan et al. 2013; Tan et al. 2014; Shaffer et al. 2017; Li et al. 2018; Liu et al. 2018; Coletta et al. 2021). Genetic variation in *WNT10A* can lead to similar phenotypic effects through the non-canonical Wnt signaling pathway (Zhang et al. 2009; Cluzeau et al. 2011). Major disruption of the ectodysplasin and Wnt signaling pathways underlie the ∼200 clinically distinct syndromes categorized as Ectodermal Dysplasia (Cluzeau et al. 2011).

When it comes to teeth, fur, sebaceous glands, and external ear structures (all influenced by the ectodysplasin pathway (Sadier et al. 2014; Archambeault et al. 2020)), there have been particularly dramatic changes in the mammalian lineages that moved into aquatic niches: external ear structures decreased in size or were lost completely, highly specialized tooth forms arose such as tusks in walruses, the narwhal “horn,” peg teeth in dolphins and killer whales, and loss of teeth in baleen whales (Hillson 2005: 200). Mammary gland form and function are also altered in the mammals who engage in aquatic lactation or fasting lactation (Oftedal 1997; Riet-Sapriza 2019). Cetaceans have mammary gland “slits” while pinnipeds have very streamlined, spread out mammary glands (Rommel et al. 2007). Fur and loss of fur, such as in walruses, manatees and cetaceans, is also notable (Espregueira Themudo et al. 2020). The sea otter has the most dense fur of all mammals species, entirely relying on its fur for insulation, in contrast to other aquatic mammals that have thick pads of blubber (Williams et al. 1992). There is also notable molting among aquatic mammals, for example, Hawaiian monk seals and elephant seals undergo a “catastrophic molt” in which they shed their fur and a layer of epidermis (Richard et al. 2014 Jan 1; de Kock et al. 2021). All these extreme phenotypic traits are potentially the result of variation in and selection on the activity of the ectodysplasin pathway.

We hypothesize that functional variation in the ectodysplasin pathway was involved in the adaptation of ectodermal structures of mammalian lineages that moved into the aquatic environment. Our approach follows the strategy employed by previous studies focused on detecting positive selection, gene-trait coevolution, and convergence across different species (e.g., (Montgomery and Mundy 2012 Sep; Muntane et al. 2018; Burskaia et al. 2021; Farré et al. 2021 Jul; Kanamori et al. 2021)) and, more specifically, among aquatic mammals (e.g., (Hammond et al. 2012; Foote et al. 2015; Marcovitz et al. 2019; Yuan et al. 2021)).

**Fig. 1:**
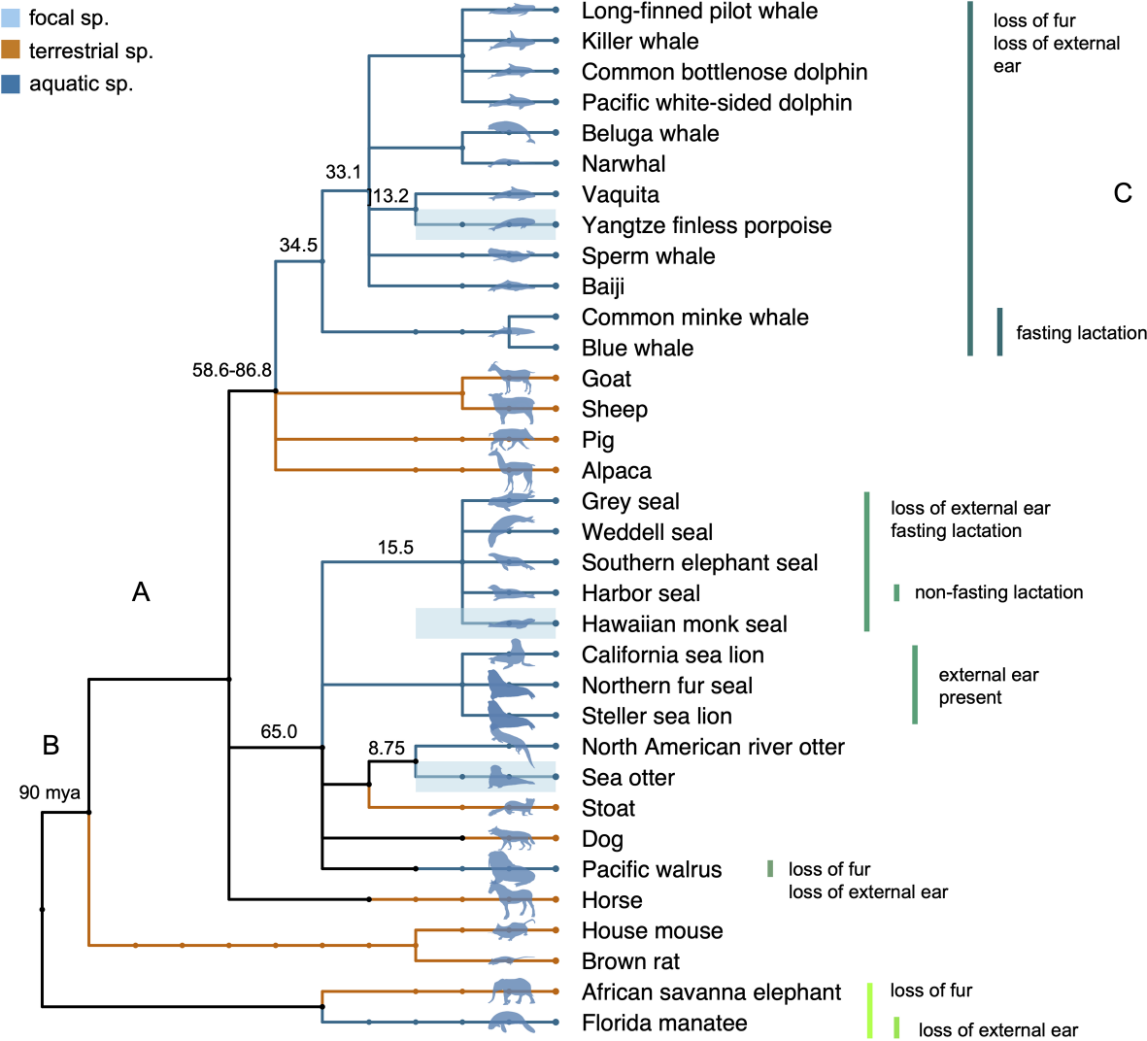
(A) Common tree exhibiting evolutionary relationships between species included in the dataset. Aquatic and semi aquatic species branches colored in blue, terrestrial species colored in orange. (B) Divergence times (in million years) noted in black. (C) Convergent traits between species marked with green bars.

We analyzed orthologous coding sequences for four major genes (*EDA*, *EDAR*, *EDARADD*, and *WNT10A*) and a suite of phenotypic traits associated with these genes for 34 species across 18 mammalian families (Table 1, Fig. 1) to test three hypotheses:

1. The ectodysplasin and non-canonical Wnt pathways evolved under positive selection in aquatic mammals as they adapted to the novel environment posed by water.
2. Ectodysplasin-related traits (primarily revealed from human genome-wide association studies, GWAS, and mouse developmental studies) coevolved with genetic variation in *EDA*, *EDAR*, *EDARADD,* and/or *WNT10A* genes.
3. Aquatic mammals from different orders experienced convergent evolution in the ectodysplasin and non-canonical Wnt pathways during their adaptation to a similar environment.

**Table 1:** List of aquatic and non-aquatic mammal species with their taxonomic classification and phenotypic trait values. Duration of lactation in days, maximum number of adult teeth, mean number of deciduous teeth, and ear size (maximum external ear length divided by the crown-rump length).

Group	Family	Common name	Scientific name	Duration of lactation	Adult teeth	Deciduous teeth	Ear size^a^
Pinnipeds	Odobenidae	Pacific Walrus	*Odobenus rosmarus*	730^1^	26^23^	28^24^	0
	Otariidae	Northern fur seal	*Callorhinus ursinus*	106.5^1^	37^24^	22^26^	0.22
		Steller sea lion	*Eumetopias jubatus*	349.5^1^	40^24^	24^27^	0.14
		California sea lion	*Zalophus californianus*	274^1^	42^24^	0^24^	0.14
	Phocidae	Grey seal	*Halichoerus grypus*	16^1^	34^24^	0^28^	0
		Harbor or common seal	*Phoca vitulina*	35^1^	34^24^	26^29^	0
		Weddell seal	*Leptonychotes weddellii*	38.5^1^	34^24^	32^30^	0
		Southern elephant seal	*Mirounga leonina*	23^1^	34^24^	16^31^	0
		Hawaiian monk seal	*Neomonachus schauinslandi*	42^1^	32^24^	0^24^	0
Cetaceans	Physteridae	Sperm whale	*Physeter catodon*	897.5^2^	60^24^	0^24^	0
	Monodontidae	Beluga whale	*Delphinapterus leucas*	639^2^	10^24^	0^24^	0
		Narwhal	*Monodon monoceros*	547.5^3^	4^24^	0^24^	0
	Delphinoidea, Phocaenidae	Vaquita	*Phocoena sinus*	274^4^	108^24^	0^24^	0
		Yangtze finless porpoise	*N.* *asiaoeorientalis*	183^5^	108^24^	0^24^	0
	Lipotidae	Baiji (Chinese river dolphin)	*Lipotes vexillifer*	365.5^6^	144^6^	0^24^	0
	Delphinidae	Common bottlenose dolphin	*Tursiops truncatus*	1642.5^7^	104^24^	0^24^	0
		Killer whale	*Orcinus orca*	547.5^8^	40^24^	0^24^	0
		Long-finned pilot whale	*Globicephala melas*	671^2^	44^24^	0^24^	0
		Pacific-whitesided dolphin	*Lagenorhynchus obliquidens*	198.5^9^	180^24^	0^24^	0
	Balaenopteridae	Blue whale	*Balaenoptera musculus*	198.5^9^	0	0^24^	0
		Common minke whale	*Balaenoptera acutorostrata*	167.5^2^	0	0^24^	0
Otters	Mustelidae	North American River otter	*Lontra canadensis*	105.5^10^	38^24^	26^24^	0.12
		Sea otter	*Enhydra lutris kenyoni*	183^11^	38^24^	26^32^	0.14
Manatee	Tichechidae	Florida manatee	*Trichechus manatus latirostris*	560^12^	28^24^	4^24^	0
Non-aquatic	Canidae	Dog	*Canis lupus familiaris*	70^13^	42^24^	28^24^	0.49
	Equidae	Horse	*Equus caballus*	168^14^	42^24^	24^24^	0.54
	Bovidae	Goat	*Capra hircus*	167^15^	32^25^	20^25^	0.62
		Sheep	*Ovis aries*	165^16^	32^16^	20^26^	0.44
	Muridae	House mouse	*Mus musculus*	23^17^	16^24^	0^24^	0.57
		Brown rat	*Rattus norvegicus*	42^18^	16^24^	0^24^	0.43
	Suidae	Wild boar	*Sus scrofa*	91.5^19^	40^24^	28^24^	0.55
	Camelidae	Alpaca	*Vicugna pacos*	91^20^	32^24^	20^24^	0.61
	Elephantidae	African savanna elephant	*Loxodonta africana*	1752^21^	12^24^	14^24^	0.9
	Mustelidae	Stoat	*Mustela erminea*	61^22^	38^24^	28^33^	0.25

a The ear/crown-rump length ratio was estimated from three photographs of the animal from lateral view. We measured the ear and cranial lengths and then report the mean of this ratio in Table 1. Each photograph was from a different female individual of the species to account for differences in sexual dimorphism.

1 (Riet-Sapriza, 2019).

2 (Oftedal, 1997).

3 (Hay, 1984).

4 (Taylor et al., 2019).

5 (Chen et al., 2018).

6 (Nowak and Walker, 2003).

7 (West et al., 2007).

8 (SeaWorld Parks & Entertainment, 2021).

9 (Ferrero and Walker, 1996).

10 (Dronkert-Egnew, 1991).

11 (Chinn et al., 2018).

12 (Physiological Ecology and Bioenergetics Lab, 2021).

13 (Mech, Barber-Meyer and Erb, 2016).

14 (Oftedal, Hintz and Schryver, 1983).

15 (Findeisen et al., 2021).

16 (Berger, Mikolayunas and Thomas, 2010).

17 (König and Markl, 1987).

18 (Miller, 1911).

19 (Marsan and Mattioli, 2013).

20 (Tibary et al., 2014).

21 (Smith and Buss, 1973).

22 (Braun, 1994).

23 (Kryukova, 2012).

24 (Hillson, 2005, p. 200).

25 (Extension Foundation. US Department of Agriculture, 2019).

26 (Aalderink et al., 2015).

27 (Marine Mammal Anatomy & Pathology Library, 2018).

28 (Kierdorf et al., 2019).

29 (Kahle et al., 2018).

30 (Thomas and Terhune, 2009).

31 (Briggs, 1974).

32 (Timm-Davis, DeWitt and Marshall, 2015).

33 (He, Friede and Kiliaridis, 2002).

## Methods

### Protein and nucleotide coding sequences

Orthologous coding sequences for *EDA*, *EDAR*, *EDARADD*, and *WNT10A* genes were downloaded from the National Center for Biotechnology Information (NCBI) for a range of aquatic mammals (including pinnipeds, cetaceans, otters, and manatee) and a set of non-aquatic mammals, using the following pipeline. We performed a BLAST search using the longest protein isoform of the human sequence as a query and retrieved the protein and nucleotide coding sequences with the lowest e-value scores and highest identity (Table S1). For the *EDARADD* gene, orthologs for the human isoform B were analyzed in order to compare the maximum number of sequences, since isoform A has been repeatedly lost during mammalian evolution (Pantalacci et al. 2008; Sadier et al. 2015: 201). Protein sequences were aligned using MAFFT v7 (Katoh and Standley 2013) and trimAL (Capella-Gutiérrez et al. 2009) was used to calculate protein identity between each pair of sequences. Protein sequences showing an average sequence identity lower than 60% might be incorrectly annotated and were discarded (Muntane et al. 2018) (Table S2). Multiple sequence alignments for the nucleotide coding sequences were computed with the codon-aware software tool PAL2NAL (Suyama et al. 2006) from the previously obtained protein alignments. Maximum likelihood phylogenetic trees were built with the codon-aligned nucleotide sequences using RaxMLv8.2.4 (Stamatakis 2014).

### Detecting positively selected sites among lineages

We used the branch-site models implemented in PAML v4.8 (Yang 2007) to assess the selective pressure (ω = *d*_N_/*d*_S_) and detect positively selected sites among aquatic mammal lineages (i.e., foreground branches) for our genes of interest. Tests were performed for each aquatic group (pinnipeds, cetaceans, otters, and manatee) and for each aquatic species individually. In each test, we ran the alternative branch-site model, which allows the ω ratio to be higher than 1 in some sites in the foreground branches, and the null model, which imposes a ω ratio ≤1 for all sites in all branches. We compared both models using a likelihood ratio test (LRT) and assessed the significance of the alternative through a chi squared test (Yang 2007). As a complementary approach, we ran the aBSREL (adaptive Branch-Site Random Effects Likelihood) method (Smith et al. 2015) from the HyPhy v2.5 package (Pond et al. 2005), setting as foreground all branches leading to the aquatic species, to detect whether a proportion of sites in these lineages are under positive selection (ω ratio > 1). Although both the branch-site model in PAML v4.8 (Yang 2007) and the aBSREL method (Smith et al. 2015) are designed to detect positive selection, we also ran the RELAX method (Wertheim et al. 2015) from the HyPhy v2.5 package (Pond et al. 2005) to discard a relaxation in purifying selection in those lineages with sites evolving under positive selection. This method specifically compares the strength of selection (positive or negative), parameter *k*, between the test (or foreground) and reference branches, testing whether selection has been intensified or relaxed (Wertheim et al. 2015).

Positively selected sites along the foreground lineages were identified with the Bayes empirical Bayes (BEB) inference (Yang et al. 2005) from PAML v4.8 (Yang 2007) and the FEL (Fixed Effects Likelihood) method (Kosakovsky Pond and Frost 2005) implemented in HyPhy v2.5 package (Pond et al. 2005). To reduce false-positive signals, sites considered significantly under positive selection must meet the following criteria: BEB posterior probabilities > 95%, FEL *P*-values < 0.05, and there was an amino acid change between the foreground and background lineages.

### Predicting the functional impact of amino acid changes

The potential impact of amino acid substitutions found in positively selected sites was assessed using four functional annotation scores, including: (i) PROVEAN (Protein Variation Effect Analyzer) with the NCBI nr database (Choi et al. 2012; Choi and Chan 2015), which is an alignment-based score that compares the protein sequence similarity with and without the amino acid substitution; (ii) PolyPhen2 (Adzhubei et al. 2010), which leverages sequence-based and structure-based information; (iii) MAPP score (Stone and Sidow 2005), which infers the impact of the substitution based on the amino acid physicochemical properties; and (iv) SIFT score (Kumar et al. 2009; Sim et al. 2012) with the UniProt-SwissProt database, which examines the sequence homology and conservation of amino acid properties. For PROVEAN, PolyPhen2, and SIFT scores, the human protein was used as reference. An amino acid substitution was considered to have a functional impact when at least three scores predicted a deleterious effect. The EDA protein structure model was generated using the SWISS-MODEL server (Waterhouse et al. 2018) using as a template the human EDA model predicted in AlphaFold (Jumper et al. 2021) (model identifier: AF-Q92838-F1) and Mol* Viewer was used to visualize the resulting 3D structures (Sehnal et al. 2021).

### Gene-trait coevolution

Phylogenetically generalized least squares (PGLS) regression analyses were conducted to test the correlation between gene and trait evolution, while controlling for the autocorrelation among species due to phylogeny (Symonds and Blomberg 2014). Gene evolution was inferred from the root-to-tip ω values for each gene and species (Montgomery and Mundy 2013; Muntane et al. 2018). These values were obtained using the free-ratio branch model implemented in PAML v4.8, which estimates a specific ω ratio for each branch (Yang 2007). As trait values, we included a series of continuous variables related to ectodermally-derived structures (Table 1). PGLS analyses were run using the Brownian motion method from the R package *nmle* (Pinheiro et al. 2021). Trait values and root-to-tip ω ratios were log_10_ transformed in all regressions. As previously suggested, species with absolute standardized residuals > 3 were considered outliers and removed from the analysis (Jones and Purvis 1997; Muntane et al. 2018; Farré et al. 2021 Jul).

### Identifying convergent amino acid evolution

To identify convergent amino acid changes, we applied the “Profile Change with One Change” (PCOC) method, which determines whether a convergent shift substitution has occurred on those branches sharing the same trait or phenotype (i.e., convergent nodes) (Rey et al. 2018). PCOC combines two tests to identify convergence: ProfileChange (PC) compares the amino acid frequencies for each site between the convergent and the ancestral nodes and OneChange (OC) determines whether the amino acid substitutions co-occur with the phenotypic changes. Particularly, convergence was tested for aquatic adaptation in mammals, including and excluding otter species, as these adapted more recently to the aquatic environments (Jefferson et al. 2015; Yuan et al. 2021). In addition, we also tested for convergence in a set of ectodysplasin-related traits among aquatic mammals (Fig. 1). Convergent amino acid shifts were considered in those sites where PCOC and OC posterior probabilities were higher than 99% and PC posterior probabilities higher than 90% (Burskaia et al. 2021).

## Results

### Signatures of positive selection in the ectodysplasin pathway in three aquatic lineages

Our estimates of the ω rate across sites on aquatic mammals (branch-site models) using two independent methods (codeml in PAML and aBSREL in HyPhy package) detected positive selection for two genes in the ectodysplasin pathway (*EDA* and *EDAR*) in three taxa (the Hawaiian monk seal, the sea otter, and the Yangtze finless porpoise).

**Fig. 2:**
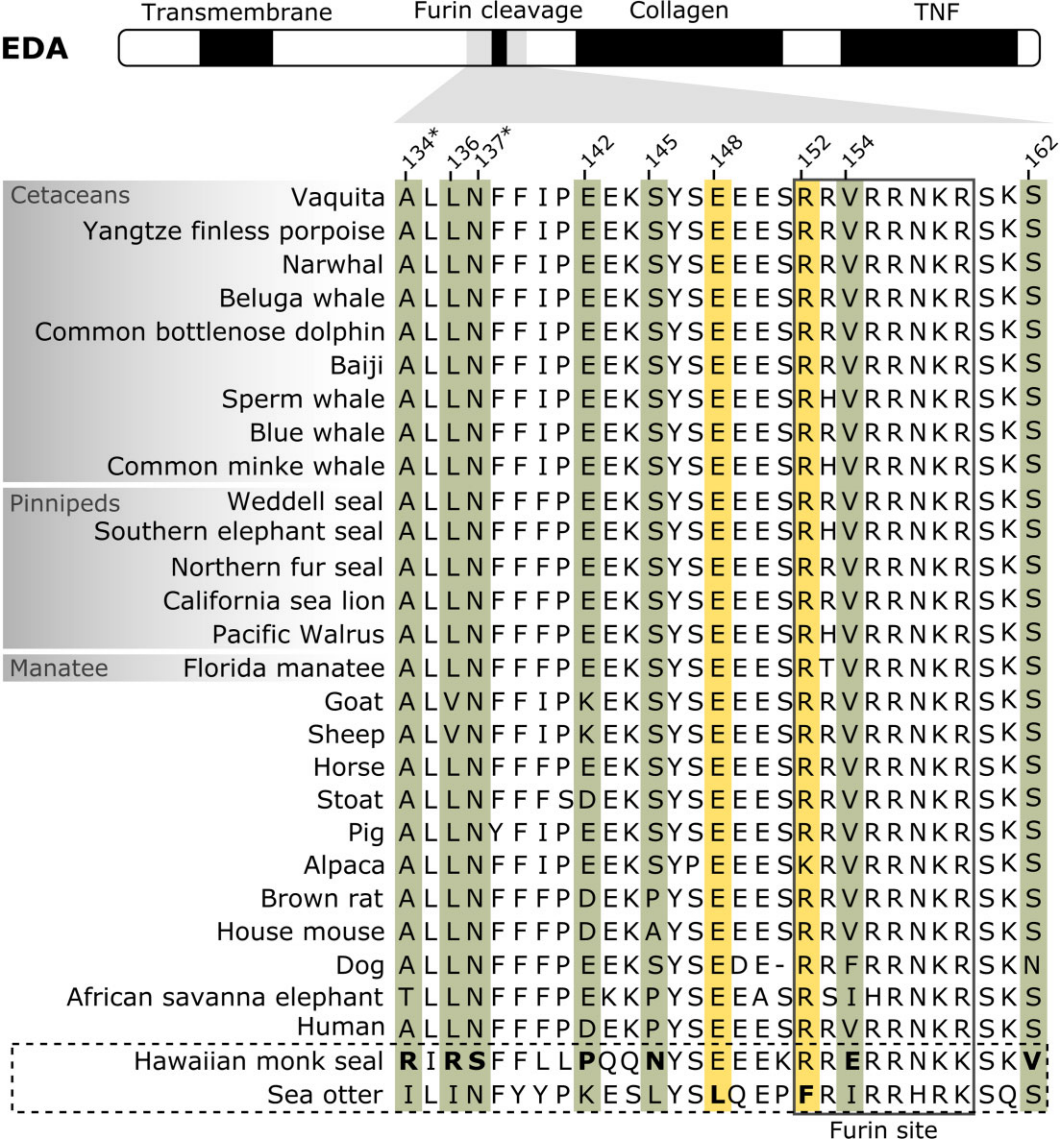
EDA protein multiple sequence alignment around the region with positively selected sites. Top diagram shows EDA protein domains (He et al. 2018). Aquatic mammals are shown within gray boxes and the aquatic species with positively selected sites are shown in the bottom within a dashed black box. Selected sites in the Hawaiian monk seal and sea otter are in bold and shadowed in green and yellow, respectively. Amino acid substitutions predicted to have a functional impact are marked with an asterisk. Consensus furin cleavage site (Chen et al. 2001) is highlighted with a black box within the alignment. The position for each selection site is given relative to the human protein sequence.

**Fig. 3:**
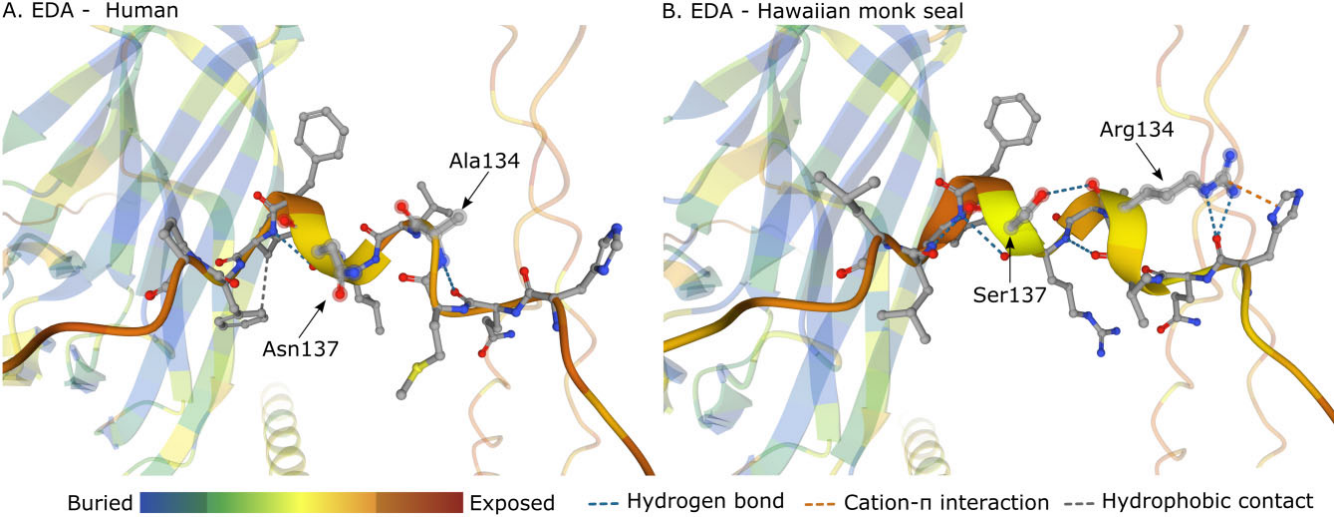
Comparative analysis of the 3D structure of the human (A) and Hawaiian monk seal (B) EDA proteins, with a focus on the positively selected sites region. Color scale depicts relative accessible surface area estimations per residue. Dashed lines represent non-covalent interactions between the residues in 134 and 137 positions and nearby sites.

The Hawaiian monk seal (*Neomonachus schauinslandi*) shows positively selected sites in the coding sequence of *EDA* gene, supported by statistically significant likelihood ratio (LR) tests from both codeml and aBSREL analyses (Table 2). Most of the sites are evolving under purifying selection (ω < 1) or neutrally (ω = 1) in all branches, yet around 9% of sites appear to be under positive selection (ω > 1) in the Hawaiian monk seal compared to the rest of branches (Table 2). In addition, RELAX method from HyPhy inferred a selection intensification in the Hawaiian monk seal lineage (*k* = 19.49, *P*-value < 0.001, LR = 62.93), suggesting these results are not driven by a relaxation of purifying selection. Focusing on the selection sites, there are seven amino acid substitutions with strong evidence of being under positive selection in this species and all of them are located around the furin cleavage region (Table 3, Fig. 2). While most of the substitutions appear to have a neutral effect on the protein, two of them are predicted to be deleterious and thus functionally relevant (A134R and N137S) (Table 3, Table S3). To further explore the impact of these substitutions, the Hawaiian monk seal EDA protein structure was modeled and compared to the human EDA model (Fig. 3). Notable differences are observed around the region with positively selected sites: a larger α-helix conformation is predicted; modifications in the non-covalent interactions (including a hydrogen bond between the mutated Arg134 and Ser137 and a cation-π interaction between Arg134 and His130); and a generally lower accessible surface area is inferred for the whole region (Fig. 3). Note that the confidence of the model on which these results are based is not high, and therefore should be read with caution: the GMQE (Global Model Quality Estimate) of the SWISS-MODEL the Hawaiian monk seal prediction is 0.46 and the per-residue confidence scores (pLDDT) of the AlphaFold predicted human model are between 45 and 50.

**Table 2:** Branches with positively selected sites for each gene. Branch-site in codeml PAML and aBSREL in HyPhy results are shown. LRT between the alternative and null models. Parameter estimates of the alternative model showing the dN/dS (ω) and proportion (p) of sites under each site class.

		Branch-site model in codeml PAML		aBSREL in HyPhy	
Gene	Branch	LRT and *P*-value	Parameter estimates	LRT and *P*-value	Parameter estimates
*EDA*	Hawaiian monk seal	140.154 (*P*-val < 0.0001)	p0 = 0.861, p1 = 0.044, p2a = 0.09, p2b = 0.005; ω0 = 0.02, ω1 = 1, ω2 = 730.1	82.073 (*P*-val < 0.0001)	ω1 = 0.228 (93.1%); ω2 = 323 (6.9%)
*EDA*	Sea otter	27.357 (*P*-val < 0.0001)	p0 = 0.844, p1 = 0.101, p2a = 0.05, p2b = 0.006; ω0 = 0.005, ω1 = 1, ω2 = 999	42.953 (*P*-val < 0.0001)	ω1 = 0.168 (93.9%); ω2 = 100,000 (6.1%)
*EDAR*	Yangtze finless porpoise	128.873 (*P*-val < 0.0001)	p0 = 0.859, p1 = 0.051, p2a = 0.085, p2b = 0.005; ω0 = 0.067, ω1 = 1, ω2 = 999	161.5480 (*P*-val < 0.0001)	ω1 = 1.00 (90.6%); ω2 = 100,000 (9.4%)

**Table 3:** Analysis of the amino acid substitutions in positively selected sites. Evidence of positive selection from BEB posterior probabilities in branch-site PAML and *P*-values from FEL HyPhy analysis. The functional impact of the substitutions was assessed using four scores (PROVEAN, MAPP, PolyPhen2, and SIFT) and considered deleterious when the prediction was supported by at least three predictions. See extended results in Table S2. Amino acid substitutions position relative to the human protein sequence.

A. *EDA* positively selected substitutions in Hawaiian monk seal (*N.schauinslandi*)			
	**Evidence of positive selection**		
**Substitution**	**BEB Post.Prob.**	**FEL** ***P*** **-values**	**Functional prediction**
A134R	1.000	0.016	Deleterious
L136R	1.000	0.001	Neutral
N137S	1.000	0.014	Deleterious
D142P	1.000	0.005	Neutral
P145N	1.000	<0.001	Neutral
V154E	1.000	0.008	Neutral
S162V	0.999	0.012	Neutral
B. *EDA* positively selected substitutions in sea otter (*E.lutris*)			
	**Evidence of positive selection**		
**Substitution**	**BEB Post.Prob.**	**FEL** ***P*** **-values**	**Functional prediction**
E148L	0.999	0.013	Neutral
R152F	0.995	0.004	Neutral
C. *EDAR* positively selected substitutions in Yangtze finless porpoise (*N.asiaoeorientalis*)			
	**Evidence of positive selection**		
**Substitution**	**BEB Post.Prob.**	**FEL** ***P*** **-values**	**Functional prediction**
H224A	0.991	<0.001	Neutral
G226W	0.992	0.002	Neutral
K227A	1.000	0.015	Deleterious
V229G	0.984	0.002	Neutral
E230R	1.000	<0.001	Deleterious
S234T	0.995	0.003	Neutral
K235P	0.980	0.022	Neutral
D236P	0.999	<0.001	Neutral
E237R	1.000	0.024	Neutral
E238L	1.000	<0.001	Deleterious
K239A	1.000	0.018	Deleterious
K240P	1.000	<0.001	Neutral
E241V	0.974	0.011	Neutral

The *EDA* coding sequence in the sea otter (*Enhydra lutris*) also shows evidence of positive selection in codeml and aBSREL analyses (Table 2), with around 6% of the sites evolving under positive selection in this lineage compared to the rest of the branches. The RELAX test also reports a selection intensification (*k* = 25, *P*-value = 0.006, LR = 7.65), rather than a selection relaxation. Two amino acid substitutions, located around the furin cleavage region, are inferred to be under positive selection in the sea otter sequence (E148L and R152F) (Fig. 2), although their functional impact prediction suggest these changes might be neutral to the protein (Table 3, Table S3).

**Fig. 4:**
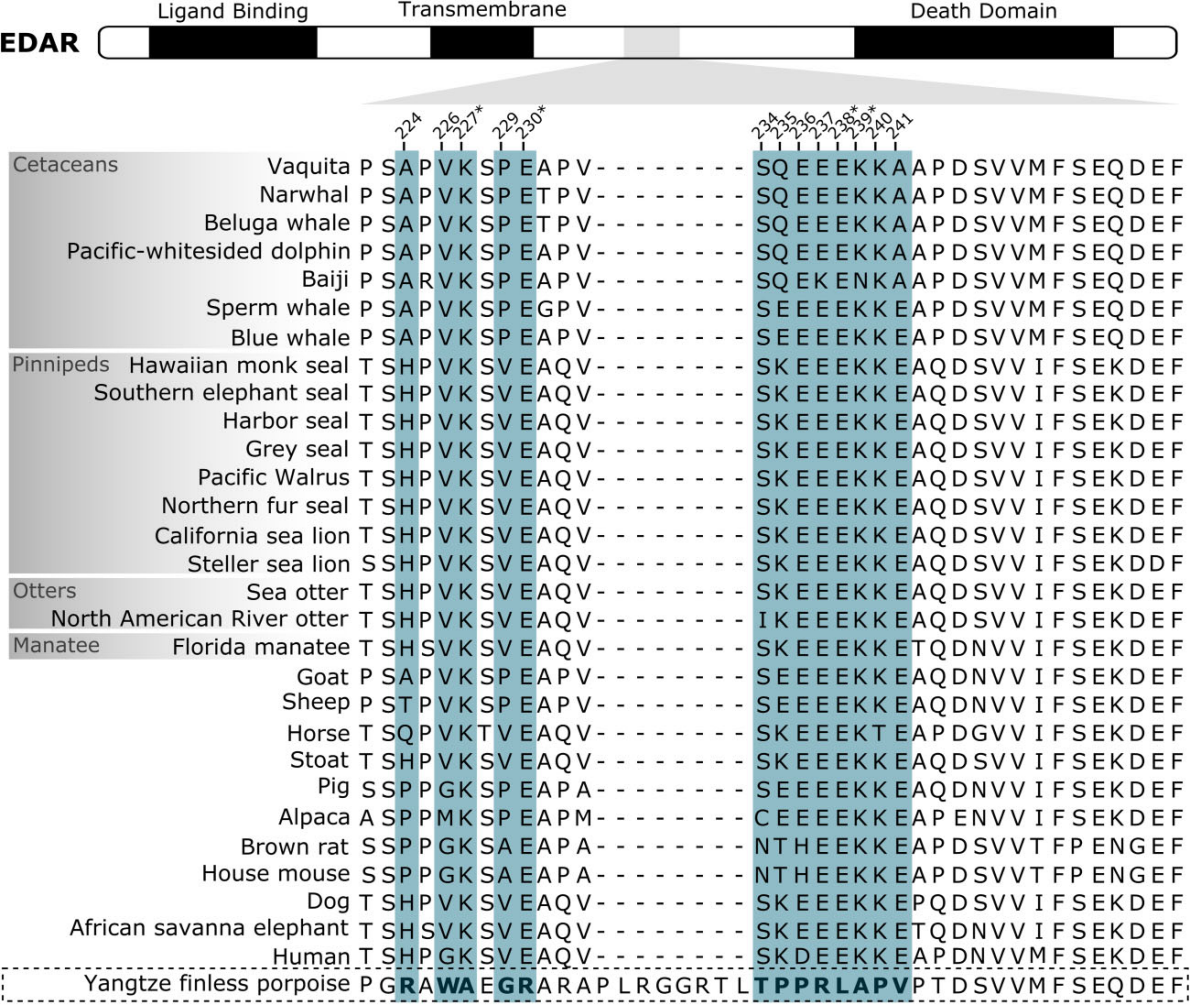
EDAR protein multiple sequence alignment around the region with positively selected sites. Top diagram shows EDAR protein domains (Sadier et al. 2014). Aquatic mammals are shown within gray boxes and the aquatic species with positively selected sites is shown in the bottom within a dashed black box. Selected sites in the Yangtze finless porpoise are in bold and shadowed in blue. Amino acid substitutions predicted to have a functional impact are marked with an asterisk. The position for each selection site is given relative to the human protein sequence.

Lastly, the branch leading to the Yangtze finless porpoise (*Neophocaena asiaoeorientalis*) shows signatures of positive selection in the *EDAR* sequence. Both codeml and aBSREL analyses are statistically significant and estimate that 9% of coding sites in this branch are under positive selection, compared to the rest of branches (Table 2). In order to assess whether these results are due to a relaxation of purifying selection, RELAX method was run evidencing the opposite pattern, a selection intensification in this branch (*k* = 50, *P*-value < 0.001, LR = 123.87). All 13 selection sites are found in the intracellular region of EDAR protein, between the transmembrane and the death domains, surrounding an insertion of eight residues only found in the Yangtze finless porpoise (Fig. 4). Regarding the impact of the amino acid substitutions, four mutations are predicted to be functionally relevant (Table 3, Table S3) and, interestingly, all involve a change from a charged residue (conserved in all species) to either an uncharged one (i.e., K227A, E238L, and K239A) or to an oppositely charged amino acid (i.e., E230K).

We did not detect a signal of positive selection on any aquatic branch of *EDARADD* or *WNT10A* coding sequences, suggesting these genes are evolving under neutral or purifying evolution.

### Associations between gene evolution and ectodermally-derived phenotypic variation

We next examined the association between the ectodysplasin pathway gene evolution (root-to-tip ω values) and the phenotypic variation found in ectodermally-derived structures, through a PGLS approach. Four continuous variables were tested as trait values, including: mean duration of lactation in days, maximum number of adult teeth, mean number of deciduous teeth, and the maximum external ear length divided by the crown-rump length (Table 1). Each analysis was conducted grouping all species together and considering aquatic and non-aquatic mammals independently to assess whether these groups exhibit different gene-trait coevolution patterns. We found no significant association between gene evolution and duration of lactation, number of adult teeth, or number of deciduous teeth (Table 4). Regarding ear size, only one gene, *EDARADD*, shows a marginally significant association when grouping all species together (uncorrected *P*-value = 0.03, slope = 1.181) (Table 4), which might suggest the evolution of this gene is involved in variation in ear size across mammals, similar to the effects of *EDAR* in humans (Adhikari et al. 2015).

**Table 4:** Phylogenetically controlled regression analyses between root-to-tip ω ratios and a set of continuous traits. Tests performed including all species, only aquatic (with and without otters) and only terrestrial. For each analysis, t-stat, *P*-value and slope are shown. NA values are shown when less than three species were available for the regression analysis.

		All			Non-aquatic			Aquatic			Aquatic without otters		
Trait	Gene	t-stat	*P*-val	slope	t-stat	*P*-val	slope	t-stat	*P*-val	slope	t-stat	*P*-val	slope
Duration of lactation	*EDA*	0.465	0.646	0.122	–0.061	0.953	–0.011	0.494	0.629	0.233	0.483	0.637	0.251
	*EDAR*	0.057	0.955	0.221	1.804	0.109	0.225	0.012	0.991	0.403	0.017	0.987	0.675
	*EDARADD*	–1.865	0.076	–2.749	–0.916	0.395	–0.524	–1.805	0.093	–4.582	–1.739	0.106	–4.595
	*WNT10A*	0.288	0.775	0.146	0.897	0.396	0.218	–0.154	0.879	–0.165	–0.400	0.694	–0.524
Number of adult teeth	*EDA*	–0.457	0.652	–0.237	–1.099	0.304	–0.066	–0.509	0.619	–0.583	–0.519	0.613	–0.679
	*EDAR*	0.266	0.792	0.113	0.581	0.577	0.030	0.622	0.543	2.541	0.630	0.539	2.964
	*EDARADD*	0.783	0.442	1.372	–0.513	0.626	–0.148	1.030	0.322	3.346	0.993	0.341	3.364
	*WNT10A*	–0.033	0.973	–0.016	–1.718	0.124	–0.132	0.199	0.844	0.214	0.222	0.827	0.297
Number of deciduous teeth	*EDA*	–0.375	0.714	–0.040	–0.330	0.752	–0.014	–0.550	0.611	–0.178	–1.176	0.324	–0.477
	*EDAR*	–0.803	0.436	–0.494	–1.224	0.267	–0.564	–0.314	0.764	–0.287	0.013	0.990	0.018
	*EDARADD*	–0.614	0.556	–0.138	–0.243	0.819	–0.063	–0.773	0.520	–0.501	–0.446	0.733	–0.421
	*WNT10A*	–0.610	0.552	–0.076	–1.294	0.243	–0.072	–1.024	0.346	–0.361	–1.722	0.160	–0.960
Ear/crown-rump length ratio	*EDA*	–0.154	0.880	–0.019	–0.072	0.945	–0.007	–0.153	0.904	–0.083	NA	NA	NA
	*EDAR*	–0.392	0.701	–0.048	–0.466	0.654	–0.037	–0.594	0.595	–1.240	–0.465	0.723	–1.419
	*EDARADD*	2.572	**0.030**	1.181	1.485	0.188	0.751	–0.565	0.673	–0.547	NA	NA	NA
	*WNT10A*	–0.549	0.592	–0.126	0.180	0.862	0.022	0.093	0.931	0.099	0.185	0.883	2.585

### No evidence for convergent amino acid substitutions

The adaptation of mammals to the aquatic environment was further explored by searching for patterns of convergent evolution in the ectodysplasin pathway. Within the range of aquatic adaptations, there are phenotypic traits related to ectodermally-derived structures that are shared among some aquatic mammals. Thus, convergence at the genomic level was tested for the following traits in these mammals: aquatic adaptation, fasting lactation strategy, loss of external ear, and loss of fur (Fig. 1). However, we did not find any evidence of convergent amino acid shifts in the tested genes for any of the shared traits as we assessed them among aquatic mammals (Table S4–S7).

## Discussion

The present study is focused on aquatic mammals, and it is important to mention that, according to the International Union for Conservation of Nature (IUCN), 25% of these species are threatened and listed as “Critically Endangered,” “Endangered,” or “Vulnerable” (Polidoro et al. 2008 Jan). Our analyses are the first to explore the role that the ectodysplasin pathway played in mammalian adaptation to aquatic environments. We tested three hypotheses using the coding sequences of three genes in the ectodysplasin pathway (*EDA*, *EDAR*, and *EDARADD*) and the similarly-functioning *WNT10A* across 34 species from 18 mammalian families, including the aquatic families Odobenidae, Phocidae, Otariidae, Phocaenidae, Delphinidae, Monodontidae, Physteridae, Lipotidae, Balaenopteridae, Enhydra, and Tichechidae. Our analyses support the first hypothesis for two aquatic species, provide only tentative support for one trait in the second hypothesis, and reject the third. We discuss these results separately in detail below.

*Hypothesis 1: The ectodysplasin and non-canonical* *Wnt* *pathways evolved under positive selection in aquatic mammals*

Our analyses revealed evidence of positive selection in the ectodysplasin pathway within three aquatic taxa: the Hawaiian monk seal (*N.schauinslandi*), the sea otter (*E.lutris kenyoni*), and the Yangtze River finless porpoise (*N.asiaoeorientalis*), likely reflecting quite distinct evolutionary histories related to the adaptation to the aquatic environments. For the three species, the positive selection signals are found in less than 10% of the protein sequence sites, which is expected given that most substitutions in a protein are neutral and thus the proportion of selected sites should be low (consistent with previous studies focused on other genes and in different species, see, for example (Park et al. 2018; Kanamori et al. 2021)).

The Hawaiian monk seal is an endangered marine mammal with very low genetic diversity due to extensive hunting in the 19th century that almost completely extirpated the species (Schultz et al. 2008). The IUCN has classified the Hawaiian monk seal as endangered since 1976, due to a range of threats, from human harassment to food insecurity from fisheries and oceanographic changes (Littnan et al. 2015). Focusing on the adaption of this species to its aquatic habitat, our study reports signals of positive selection in a proportion of sites of the *EDA* coding sequence of this species, which are not due to a relaxation of purifying selection. The positively selected substitutions are located near the furin cleavage site of the protein and some of them are predicted to have a functional impact. EDA is a transmembrane protein that binds EDAR and can activate a juxtacrine signaling (adjacent cells) or a paracrine signaling (distant cells) when it is cleaved by furin and released as a soluble ligand (Chen et al. 2001; Thomas 2002; Cui and Schlessinger 2006: 201). Mutations blocking the furin cleavage have been previously described in humans and associated to X-linked hypohidrotic ectodermal dysplasia, suggesting the EDA paracrine signaling is essential for development (Chen et al. 2001; Pääkkönen et al. 2001; Schneider et al. 2001; Kowalczyk-Quintas and Schneider 2014; Sadier et al. 2014). However, the amino acid substitutions under selection in the Hawaiian monk seal (A134R and N137S) are found upstream of the cleavage site and shown to lower the accessible surface area of the region compared to the human wild-type protein. While these substitutions do not directly impair the EDA release, the reduced solvent accessibility of the upstream flanking region might decrease the efficiency of the furin cleavage (Tian et al. 2012), which in turn might lead to a reduced EDA paracrine activity. While our analysis of a coarse set of phenotypic traits for hypothesis 2 did not yield insight as to what anatomical region may have been the target of this selection, we find it noteworthy that the Hawaiian monk seal has the lowest latitudinal range of all aquatic mammals (together with the Florida manatee), being the only extant tropic pinniped species (Donohue and Foley 2007). Our results suggest that the activity of the ectodysplasin pathway may be reduced in Hawaiian monk seals, which is the opposite of the strong positive selection for increased activity in the ectodysplasin pathway associated with the V370A variant of *EDAR* in a human population living in the Arctic during the last glacial maximum (Mou et al. 2008; Chang et al. 2009; Kamberov et al. 2013), selection hypothesized to result from the dramatically low levels of subcutaneously derived vitamin D given the extremely low levels of ultraviolet radiation at this latitude (Hlusko et al. 2018). Although the Hawaiian monk seal is not the only low latitude aquatic mammal, similar selective pressures can lead to different genetic changes (Eidem et al. 2015). Thus, these results suggest that further investigation into the relationship between latitude and the activity of the ectodysplasin pathway may be merited.

The sea otter is the only fully aquatic mustelid and has been on the IUCN Endangered list since 2000, mostly due to climate change effects (e.g., ocean acidification), oil spills, and commercial fishing nets, among other anthropogenic factors (Doroff and Burdis 2015). Ectodysplasin gene sequences are only available in NCBI for the northern sea otter (*E.lutris kenyoni*), one of the three subspecies that occupies a range from the Aleutian Islands in Alaska to the Prince William Sound and parts of northern Washington state (Doroff and Burdis 2015). Our analyses reveal two positively selected sites on the *EDA* coding sequence of this subspecies. However, both amino acid substitutions (E148L and R152F) are not predicted to have a functional impact, suggesting they might be neutral to the protein. These results are consistent with genetic drift as the main cause of the observed signals, which leads more rapidly to the loss or fixation of alleles, especially in smaller populations. In fact, *E.lutris* went through at least one bottleneck and near extirpation due to the fur trade, between 1741 and 1911 (Larson et al. 2012: 20). Previous studies report that genetic diversity in this species was severely impacted by the fur trade: modern populations of sea otters have about half their genetic heterozygosity of pre-fur trade populations, as well as less than 34% of alleles within microsatellite loci (Larson et al. 2012: 20). In addition to a reduction of diversity, genetic drift contributes to an increased differentiation between isolated populations, which is also observed in sea otters, since they appear to have maintained their population structure (Larson et al. 2012: 20). Alternatively, this result could be driven by a false positive signal, given that the proportion of positively selected sites detected (6%) is close to the 5% error rate.

The Yangtze finless porpoise is the world's only freshwater porpoise and it is classified as “Critically endangered” by the IUCN, due to fishing and harvesting aquatic resources, human intrusions, water pollution, and habitat shifting as a result of climate change (Wang et al. 2013). This species is endemic to the middle-lower Yangtze River drainage basin in eastern China (Wang et al. 2013) and shared its range with the Yangtze River dolphin (or Baiji, *Lipotes vexillifer*) before the latter went functionally extinct in 2006 (World Wildlife Fund 2021). Regarding its adaptation to water, our analyses identified signals of positive selection in the *EDAR* coding sequence of the Yangtze finless porpoise, consistent with an intensification of selection rather than a relaxation of purifying selection. Based on the impact scores, four amino acid substitutions are predicted to be functionally relevant, although we could not find any evidence of their functional role, since the selection sites are in the intracellular region outside the protein domains. A previous study on the Yangtze finless porpoise and other narrow ridged marine populations found selection in genes associated with renal function, which was related to the adaptation to the hypotonic freshwater environment (Zhou et al. 2018: 201). Interestingly, a GWAS of kidney function traits in humans revealed an association between *EDAR* variation and the urine albumin to creatinine ratio (Qian et al. 2020), suggesting that the selection found in this gene in the Yangtze finless porpoise might be also involved in the adaptation to freshwater through renal regulation. Although this signal of positive selection is only observed in the Yangtze finless porpoise and not in the Yangtze River dolphin (the other freshwater cetacean with almost identical geographic range present in our analysis), the divergence time between both species is estimated to be around 20 million years ago (Yuan et al., 2018), thus, the adaptation to freshwater might have occurred through a different mechanism in each species.

Altogether these results suggest that the ectodysplasin pathway evolved under positive selection in at least two aquatic mammalian species. This evidence of positive selection might be related to aquatic adaptation (in low latitudes or freshwater environments) or to the adaptation to other environmental features. Functional analyses and experimental validation are needed to decipher the role of the amino acid substitutions detected to be under positive selection in these taxa, regarding their impact to the protein and their phenotypic implications related to the species habitats. In addition, the present study has been performed using only one sequence per species, thus, there is a need to sequence more individuals from the same species to examine genetic variation and to determine whether these substitutions are fixed or polymorphic in these populations.

*Hypothesis 2: Ectodysplasin-related traits coevolved with genetic variation in EDA, EDAR, EDARADD, and/or WNT10A*

Besides a marginal association for ear size, we found no evidence of coevolution between gene rate evolution in *EDA*, *EDAR*, *EDARADD*, and *WNT10A* and variation in the tested ectodysplasin-related traits (duration of lactation, number of adult teeth, and number of deciduous teeth). The lack of strong statistically significant associations might be related to the way we assessed the traits; thus, we are not confident that the absence of evidence here is evidence of absence. In addition, the PGLS approach tests whether gene evolution is consistently associated with changes in the trait (i.e., across all species) and in this case, species-specific mechanisms would be missed. Alternatively, non-significant results could be driven by the low sample size, since the maximum number of species included in the analyses is 34 and previous studies pointed to sample size as a limiting factor for PGLS analyses (Muntane et al. 2018). To comprehensively understand the pleiotropic effects of the ectodysplasin pathway, future studies should address the above-mentioned limitations and extend the data collection of mammalian species with other traits associated with the ectodysplasin pathway, for example, breast density (Coletta et al. 2021), hair thickness (Fujimoto et al. 2008), etc.

*Hypothesis 3: Aquatic mammals from different orders might have experienced convergent evolution in the ectodysplasin non-canonical* *Wnt* *pathways during their adaptation to a similar environment*

Convergent evolution in protein-coding genes has been previously detected in aquatic mammals and linked to convergent phenotypic evolution for bone density (*S100A9* and *MGP* genes) (Foote et al. 2015), inner ear formation (*SMPX* gene) (Foote et al. 2015), thermal insulation (*NFIA* gene) (Yuan et al. 2021), and skin keratinization (*ABCA12* gene) (Marcovitz et al. 2019), among others. In the present study, we hypothesized that aquatic mammals might have experienced convergent evolution in the ectodysplasin and non-canonical Wnt pathways, given the importance of ectodermally-derived structures to the adaptation to water (Reidenberg 2007). However, we have not detected any convergent amino acid shift in these pathways related to aquatic adaptation or to other traits shared between some aquatic mammals. This largely negative result suggests that either aquatic mammals are not using variation in the ectodysplasin pathway to adapt, or they are using different unique coding mutations. The latter explanation is the more plausible given that the entire ectodysplasin pathway appears to be a hotspot for evolutionary change (e.g., (Sadier et al. 2014; Zhang et al. 2018; Archambeault et al. 2020; Schluter et al. 2021)) and that convergent genetic evolution leading to phenotypic convergence has been proven to be rare (Foote et al. 2015). Alternatively, aquatic adaptation might have occurred through non-coding variation in the ectodysplasin pathway, due to the important role of regulatory regions in phenotypic changes (Carroll 2008).

In summary, our study reports evidence of positive selection in the coding sequence of two ectodysplasin pathway genes (*EDA* and *EDAR*) in three endangered aquatic mammals. We hypothesize functional implications related to the adaptation to specific aquatic environments for the Hawaiian monk seal (low latitude adaptation) and the Yangtze finless porpoise (freshwater adaptation); while the signal in the sea otter appears to derive from neutral substitutions driven by an increased genetic drift. In addition, we could not identify strong evidence of gene-trait coevolution or convergent amino acid substitutions in the ectodysplasin pathway, thus, future studies including an extensive set of mammalian traits are needed to uncover the full range of phenotypic effects related to variation in these genes. Also, given the endangered status of many aquatic mammals, including the ones analyzed in the present study, we encourage the development of conservation policies and measures in order to preserve the biodiversity of marine and freshwaters environments.

## Supplementary Material

obac018_Supplemental_File

## Data Availability

The data underlying this article were accessed from National Center for Biotechnology Information (NCBI) with the accession numbers specified in Table S1. Phenotypic data were culled from the published literature cited herein.
